# Inhibition of hTERT/telomerase contributes to the antitumor activity of pristimerin in pancreatic ductal adenocarcinoma cells

**DOI:** 10.3892/or.2015.3989

**Published:** 2015-05-19

**Authors:** DORRAH DEEB, XIAOHUA GAO, YONGBO LIU, KIRIT PINDOLIA, SUBHASH C GAUTAM

**Affiliations:** 1 Department of Surgery, Henry Ford Health System, Detroit, MI 48202, USA; 2 Department of Research, Henry Ford Health System, Detroit, MI 48202, USA

**Keywords:** pristimerin, pancreatic cancer, apoptosis, hTERT, telomerase

## Abstract

Pristimerin (PM) is a promising anticancer agent that has exhibited strong antiproliferative and apoptosis-inducing activity in various types of cancer cells. In the present study, we investigated the role of telomerase in mediating the antitumor activity of PM in pancreatic ductal adenocarcinoma (PDA) cells. PM inhibited cell proliferation, arrested cells in the G1 cell cycle phase and induced apoptosis in MiaPaCa-2 and Panc-1 PDA cells. These antitumor activities of PM correlated well with the inhibition of human telomerase reverse transcriptase (hTERT), the gene that codes for the catalytic subunit of telomerase complex. Gene knockin and knockdown approaches demonstrated that hTERT regulates the response of PDA cells to PM. PM inhibited hTERT expression by suppressing the transcription factors Sp1, c-Myc and NF-κB which control hTERT gene expression. PM also inhibited protein kinase Akt, which phosphorylates and facilitates hTERT nuclear importation and its telomerase activity. These findings identified hTERT (telomerase) as a potential therapeutic target of PM for the treatment of PDA.

## Introduction

Pancreatic ductal adenocarcinoma (PDA) is almost uniformly lethal with an estimated annual number of 45,220 new cases resulting in ~38,460 annual mortalities and a 5-year survival rate of <5% (1–3). Late initial diagnosis, aggressive metastatic behavior and resistance to chemoradiotherapy render pancreatic cancer one of the most difficult malignancies to treat. Surgical resection is potentially curative in a minority of patients. However, almost 80% of the patients are diagnosed with locally advanced disease that precludes surgical intervention. Systemic gemcitabine alone or in combination with 5-FU, irinotecan and oxaliplatin (FOLFIRINOX) is the current standard of care for advanced pancreatic cancer, providing short-term symptomatic improvement with minor impact on survival (4–6). Thus, the development of novel agents targeting novel biomarkers for the treatment of pancreatic cancer is imperative.

Pristimerin (PM) is a quinonemethide triterpenoid present in various plant species in the *Celastraceae* and *Hippocrateaceae* families. PM has shown potent antiproliferative and apoptosis-inducing activity in various types of cancer cells including pancreatic cancer cells (7–11). Induction of apoptosis by PM involved the generation of reactive oxygen species (ROS), activation of caspases, mitochondrial dysfunction and the inhibition of nuclear factor κB (NF-κB), Akt and MAP kinases (12,13).

Telomeres are nucleoprotein structures present at the end of chromosomes that play an essential role in chromosomal stability and protection from end-to-end fusion (14). Telomeres shorten progressively during each cell division due to the gradual loss of the telomeric DNA sequence (15). When the telomere length becomes critically short, it triggers replicative senescence or apoptosis. Maintaining the telomere length by incorporating hexameric DNA repeats (TTAGGG) to the 3′ flanking end of DNA strands is the function of telomerase, a ribonucleoprotein complex. Human telomerase comprises the RNA template (hTERC) and RNA-dependent DNA polymerase human telomerase reverse transcriptase (hTERT) (16,17). hTERC serves as a template for hTERT-mediated telomere extension. In addition, hTERT associates with several proteins including a six-protein complex known as shelterin for proper functioning (18,19). Deregulated telomerase activity is associated with the promotion of tumorigenesis and neoplastic growth of cancer (20,21). Approximately 90% of human cancer types exhibit activated telomerase (22). hTERT expression and telomerase activity are elevated in pancreatic cancers with PDA showing significantly higher telomerase activity (23–25). The high incidence of active telomerase in PDA suggests that targeting telomerase is a promising strategy for the treatment of this disease.

In the present study, we investigated the effect of PM on the expression of hTERT and hTERT telomerase activity in the MiaPaCa-2 and Panc-1 PDA cell lines. PM inhibited hTERT mRNA, native and phospho-hTERT protein and telomerase activity. PM also downregulated proteins that regulate hTERT transcriptionally and post-translationally.

## Materials and methods

### Reagents

PM was purchased from Sigma Chemicals (St. Louis, MO, USA). Antibodies against PARP-1, Akt, p-Akt (Ser^473^), Sp1, c-Myc, NF-κB and β-actin were purchased from Santa Cruz Biotechnology, Inc. (Santa Cruz, CA, USA). Anti-hTERT and p-TERT antibodies were obtained from Abcam, Inc. (Cambridge, MA, USA). The CellTiter 96^®^ AQueous One Solution Proliferation Assay System was purchased from Promega (Madison, WI, USA). The Annexin V-FITC Apoptosis Detection Kit II was obtained from BD Biosciences Pharmingen (San Diego, CA, USA) and the TRAPeze Telomerase Detection kit was purchased from Millipore (Temecula, CA, USA).

A stock solution (100 mM) of PM was prepared in dimethyl-sulfoxide (DMSO) and test concentrations were prepared by diluting the stock solution in tissue culture medium.

### Cell lines

MiaPaCa-2 and Panc-1 PDA cell lines were obtained from the American Type Culture Collection (ATCC; Rockville, MD, USA). The two cell lines were grown in Dulbecco’s modified Eagle’s medium (DMEM) tissue culture medium (Gibco-BRL, Rockville, MD, USA) supplemented with 10% fetal bovine serum, 1% penicillin/streptomycin and 25 mM HEPES buffer. The cells were incubated at 37°C in a humidified atmosphere consisting of 5% CO_2_, 95% air and maintained by splitting the cultures twice a week.

### MTS assay

Tumor cells (1×10^4^) in 100 *μ*l of tissue culture medium were seeded in each well of 96-well plates. After a 24-h incubation to allow cells to adhere, the cells were treated with PM at concentrations ranging from 0 to 5 *μ*M. The cultures were incubated for an additional 72 h and cell viability was then determined by the colorimetric 3-(4,5-dimethylthiazol-2-yl)-5-(3-carboxymethoxyphenyl)-2-(4-sulfophenyl)-2H-tetrazo lium (MTS) assay using the CellTiter 96 AQueous One Solution Proliferation Assay System. This assay measures the bioreduction of the tetrazolium compound MTS in the presence of the electron-coupling reagent phenazine methosulfate by intracellular dehydrogenases. MTS and phenazine methosulfate were added to the culture wells, and the cultures were incubated for 2 h at 37°C. The absorbance, which is directly proportional to the number of viable cells in the cultures, was measured at 490 nm using a microplate reader.

### Cell cycle analysis

The distribution of cells in various cell cycle phases was analyzed by measuring cell DNA content. Untreated (control) (2×10^6^) or PM-treated cells were fixed in 70% ethanol overnight at 4°C. The cells were washed twice and resuspended in 0.8 ml of phosphate-buffered saline (PBS). In total, 100 *μ*l of DNAse free RNAse (500 *μ*g/ml) and 100 *μ*l of propidium iodide (PI) (500 *μ*g/ml) were added to each tube and the tubes were incubated at room temperature in the dark for 30 min. Cell DNA content was determined by flow cytometry using an Accuri C6 flow cytometer (Accuri Cytometers, Inc., Ann Arbor, MI, USA).

### Annexin V-FITC binding

Induction of apoptosis was assessed by the binding of Annexin V to phosphatidylserine, which was externalized to the outer leaflet of the plasma membrane early during induction of apoptosis. Briefly, MiaPaCa-2 and Panc-1 cells treated with PM (0–5 *μ*M) for 24 h were resus-pended in the binding buffer provided in the Annexin V-FITC Apoptosis Detection Kit II. The cells were mixed with 5 *μ*l of the Annexin V-FITC reagent, 5 *μ*l of PI and incubated for 30 min at room temperature in the dark. The stained cells were analyzed by flow cytometry.

### Western blotting

Cell lysates were prepared by CHAPS detergent lysis [1% Triton X-100 (v/v), 10 mM Tris-HCl (pH 7.5), 5 mM EDTA, 150 mM NaCl, 10% glycerol, 2 mM sodium vanadate, 5 *μ*g/ml leupeptin, 1 *μ*g/ml aprotinin, 1 *μ*g/ml pepstatin A and 10 *μ*g/ml 4-2-aminoethyl-benzenesulfonyl fluoride]. The lysates were clarified by centrifugation at 14,000 × g for 10 min at 4°C, and protein concentrations were determined by the Bradford assay. Samples (50 *μ*g) were boiled in an equal volume of sample buffer [20% glycerol, 4% SDS, 0.2% bromophenol blue, 125 mM Tris-HCl (pH 7.5) and 640 mM 2-mercaptoethanol] and separated on 10% SDS-polyacrylamide gels. Proteins resolved on the gels were transferred to nitrocellulose membranes. The membranes were blocked with 5% milk in 10 mM Tris-HCl (pH 8.0), 150 mM NaCl with 0.05% Tween-20 (PBS) and incubated with protein-specific antibodies followed by an HRP-conjugated secondary antibody. Immune complexes were visualized with an enhanced chemiluminescence detection system from Amersham Corp. (Arlington Heights, IL, USA) and protein bands were imaged.

### Transfections

For hTERT overexpression, semi-confluent cell cultures were transfected with 10 *μ*g of empty or hTERT expression plasmid (pCI-neo-hTERT) DNA using Lipofectamine Plus reagent. After transfection for 48 h, the cells were analyzed for the expression of hTERT by immuno-blotting.

For the silencing of hTERT, the cells were transfected with double-stranded siRNA-hTERT or a non-targeting siRNA sequence using a SignalSilence siRNA kit (Cell Signaling Technology, Beverly, MA, USA). Briefly, 2×10^6^ tumor cells were plated in a 60-mm Petri dish for 24 h and treated with 3 ml of transfection medium containing 20 *μ*g Lipofectamine and 100 nM siRNA for 48 h. The hTERT expression was analyzed by immunoblotting.

### Statistical analysis

Data are presented as means ± SD. The differences between the control and treatment groups were analyzed using the Student’s t-test. P<0.05 was considered to indicate a statistically significant result.

## Results

### PM inhibits proliferation and cell-cycle progression in PDA cells

**Figure 1:**
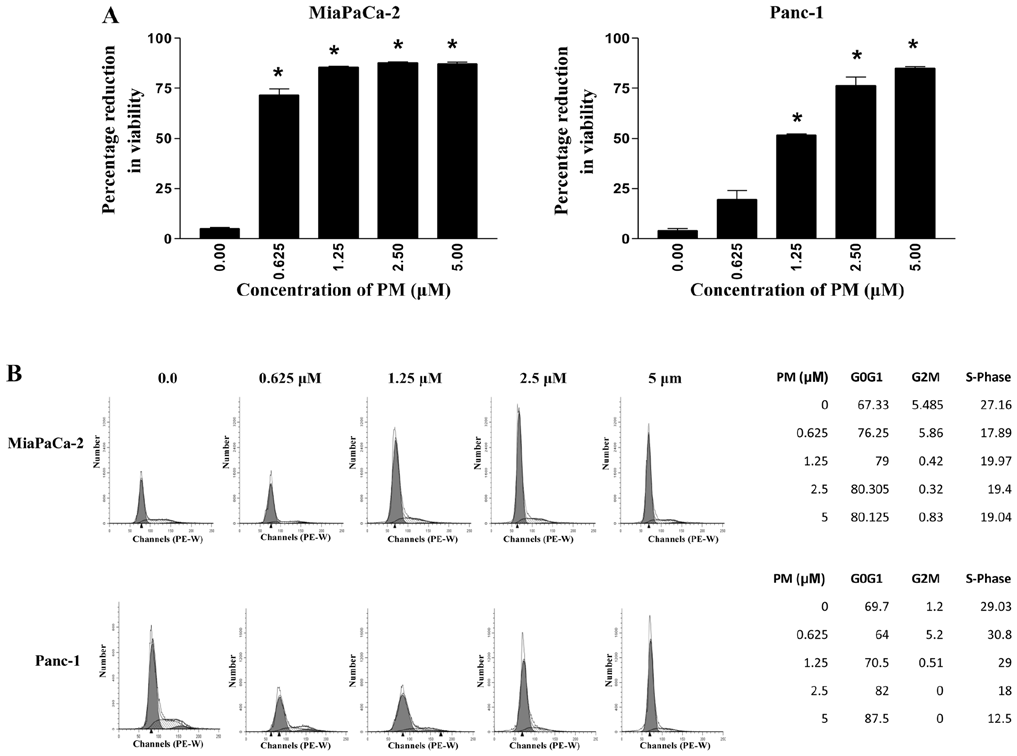
PM inhibits cell proliferation and cell cycle progression in PDA cells. (A) Effect on cell proliferation. MiaPaCa-2 and Panc-1 cells (1×10^4^) were seeded in each well of 96-well plates. After incubation for 24 h, the cells were treated with PM at concentrations ranging from 0 to 5 *μ*M for 72 h in triplicate. Cell viability was measured by the MTS assay using the CellTiter AQueous Assay System. ^*^P<0.05 compared to the control cells. (B) Effect on cell-cycle progression. MiaPaCa-2 and Panc-1 cells treated with PM (0–5 *μ*M) for 24 h were fixed and stained with PI. Cellular DNA content was analyzed by a flow cytometry and cell distribution in various cell cycle phases was analyzed. PM, pristimerin; PDA, pancreatic ductal adenocarcinoma; PI, propidium iodide.

The effect of PM on proliferation of PDA cells (MiaPaCa-2 and Panc-1 cells) was examined using the MTS assay. Briefly, the cells were treated with PM at concentrations of 0–5 *μ*M for 72 h and the viability of the cultures was determined. As shown in Fig. 1A, a significant (MiaPaCa-2, 71%) to measurable (Panc-1, 19%) reduction in viability was observed at 0.625 *μ*M PM. In the MiaPaCa-2 cells the viability reached a plateau (85–87%) at 1.25-5 *μ*M PM. On the other hand, reduction of viability in the Panc-1 cells occurred in a dose-dependent manner (51, 76 and 85% at 1.25, 2.5 and 5 *μ*M PM). Thus, PM significantly reduced the proliferation of the two PDA cell lines at PM concentrations of 1.25–5 *μ*M.

The effect of PM on cell cycle progression was then analyzed. MiaPaCa-2 and Panc-1 cells were treated with PM (0–5 *μ*M) for 24 h, stained with PI and cell DNA content of the cells was measured by flow cytometry. As shown in Fig. 1B, treatment with PM resulted in cell cycle arrest in G1-phase with a cell distribution of 67.3, 76.2, 79, 80.3 and 80.1% in MiaPaCa-2 cells and 69.7, 64, 70.5, 82 and 87.5% in Panc-1 cells at 0, 0.625, 1.25, 2.5 and 5 *μ*M PM, respectively.

### PM induces apoptosis in PDA cells

**Figure 2:**
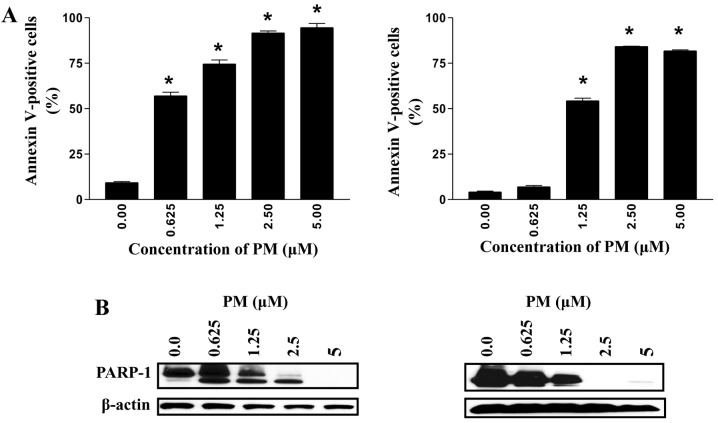
PM induces apoptosis in PDA cells. (A) MiaPaCa-2 and Panc-1 cells were treated with PM at 0–5 *μ*M for 24 h. The cells were then reacted with 5 *μ*l of Annexin V-FITC and 5 *μ*l PI for 30 min and the percentage of Annexin V-FITC binding cells was determined by flow cytometry. (B) MiaPaCa-2 and Panc-1 cells were treated with PM as above and the cell lysates were analyzed for the cleavage of PARP-1 by western blotting. Similar results were obtained in two independent experiments. PM, pristimerin; PDA, pancreatic ductal adenocarcinoma.

Whether the cell cycle arrest leads to the induction of apoptosis was investigated by measuring the binding of Annexin V-FITC and cleavage of PARP-1 in tumor cells treated with PM. Thus, MiaPaCa-2 and Panc-1 cells were treated with PM (0–5 *μ*M) for 24 h and the binding of Annexin V-FITC was determined by flow cytometry. As shown in Fig. 2, only a small percentage of untreated MiaPaCa-2 and Panc-1 cells bound to Annexin V-FITC (9–4%, respectively). By contrast, the percentage of Annexin V-FITC binding cells in the two cell lines increased in a dose-dependent manner following treatment with PM at 0.625–5 *μ*M (MiaPaCa-2, 57–91%; Panc-1, 7–81%).

The induction of apoptosis was confirmed by the cleavage of PARP-1. Thus, the tumor cells were treated with PM as described above and cleavage of PARP-1 was determined by western blotting. As shown in Fig. 2B, treatment with PM induced the cleavage of native PARP-1 (110-kDa fragment) as identified by the emergence of an 89-kDa cleaved PARP-1 fragment in the two cell lines treated with PM. The increase in Annexin V-FITC binding and the cleavage of PARP-1 demonstrated induction of apoptosis in PDA cells by PM.

### PM inhibits hTERT expression in PDA cells

**Figure 3:**
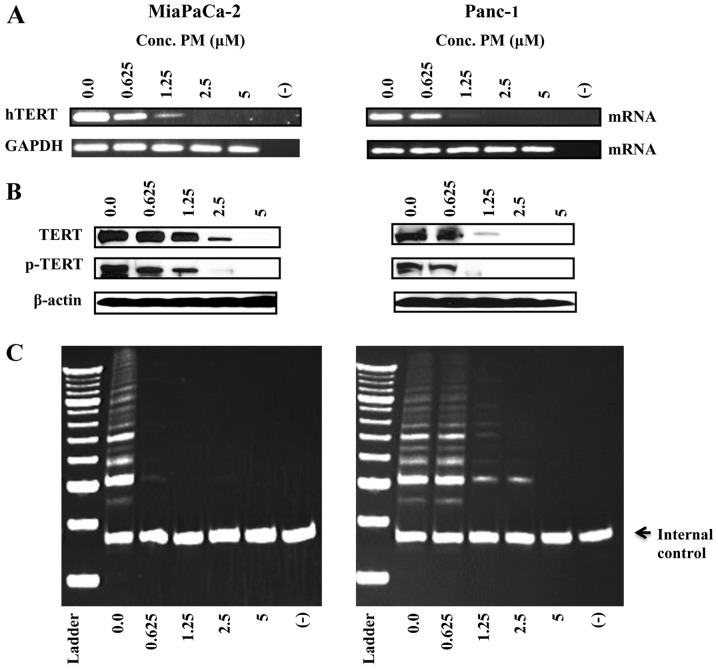
PM inhibits hTERT mRNA and hTERT protein in PDA cells. (A) Effect on hTERT gene expression. MiaPaCa-2 and Panc-1 cells were treated with PM (0–5 *μ*M) for 48 h and total cellular RNA was prepared using TRIzol reagent. Cellular RNA (1 *μ*g) was reverse transcribed using oligo(dT) primer and high fidelity reverse transcriptase. cDNA (1 *μ*l) was amplified using hTERT or GAPDH primers. Amplified products were separated on a 2% DNA agarose gel. Gels were stained with ethidium bromide and amplified DNA fragments were identified by base pair sizes. (B) Effect on hTERT protein. MiaPaCa-2 and Panc-1 cells were treated with PM and cell lysates were analyzed for hTERT and p-hTERT protein by western blotting. (C) Effect on telomerase activity. MiaPaCa-2 and Panc-1 cells treated with PM (0–5 *μ*M) for 48 h were extracted in CHAPS buffer. The telomerase activity in the cell extracts was measured by the PCR-based TRAP assay as described in Materials and methods. Negative (−), no cell extract. Each experiment was repeated at least twice. PM, pristimerin; PDA, pancreatic ductal adenocarcinoma.

Reactivation of telomerase promotes the proliferation of tumor cells. Therefore, we determined the effect PM exerts on hTERT expression and its telomerase activity. Briefly, hTERT mRNA was analyzed by RT-PCR and hTERT protein by western blotting following treatment of the tumor cells with PM. Treatment with PM at concentrations of 1.25-5 *μ*M for 48 h completely inhibited hTERT mRNA in the two cell lines (Fig. 3A). PM at these concentrations, however, had no effect on the expression of the housekeeping gene *GAPDH*.

Treatment with PM (1.25–5 *μ*M) also resulted in partial to complete reduction in the native and phosphorylated-hTERT (Ser^826^) protein in the two cell lines.

### PM inhibits telomerase activity

To determine whether PM affects hTERT telomerase activity, MiaPaCa-2 and Panc-1 cells were treated with PM (0–5 *μ*M) for 48 h and the cells were extracted in CHAP lysis buffer. The telomerase activity in the extracts was measured using the PCR-based TRAP assay. As shown in Fig. 3C, the telomerase activity in the MiaPaCa-2 cells was completely abolished even at the lowest concentration of 0.625 *μ*M PM, as determined from the complete loss of DNA laddering. Telomerase activity was also markedly reduced in Panc-1 cells at 1.25 and 2.5 *μ*M PM and completely abolished at 5 *μ*M.

### hTERT regulates the response to PM in PDA cells

**Figure 4:**
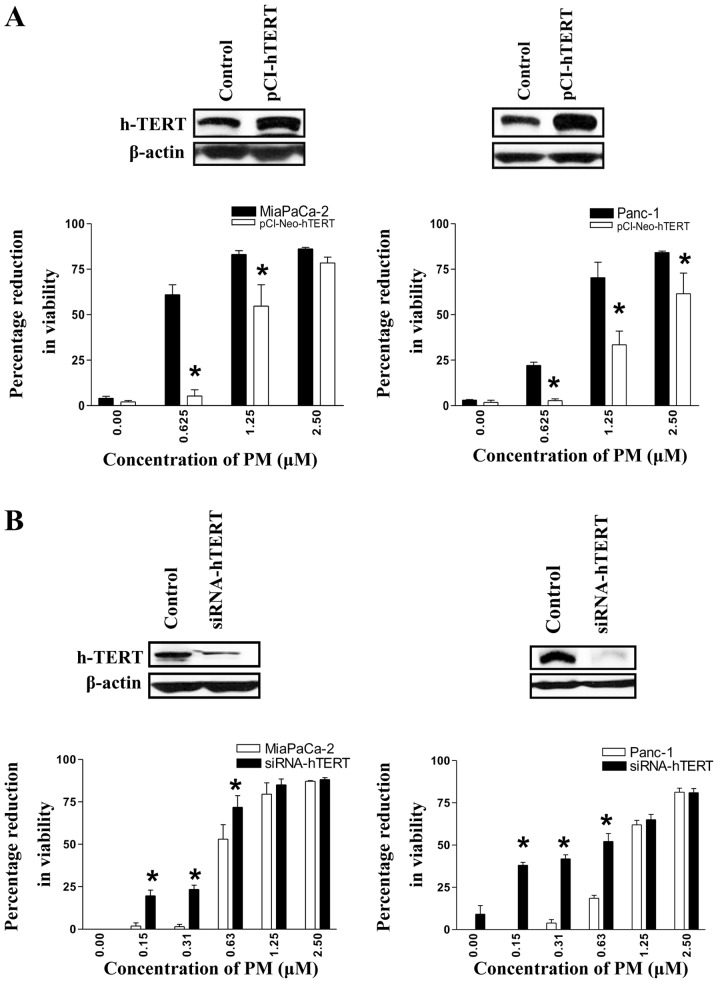
hTERT mediates response to PM in PDA cells. MiaPaCa-2 and Panc-1 cells were transfected with (A) hTERT expression vector (pCI-neo-hTERT) or (B) siRNA-hTERT for 48 h using Lipofectamine Plus reagent. hTERT levels were determined by immunoblotting (insets) and sensitivity of control and transfected cells to PM was assessed in the MTS assay. Each experiment was repeated twice. PM, pristimerin; PDA, pancreatic ductal adenocarcinoma.

To identify the involvement of telomerase in mediating the antiproliferative and apoptosis-inducing activity of PM, we genetically altered the expression of hTERT in MiaPaCa-2 and Panc-1 cells and measured their response to PM. To increase hTERT expression, the tumor cells were transfected with pCI-neo-hTERT expression vector for 48 h and the response to PM was measured in the MTS assay. As expected, the transfected cells showed higher levels of hTERT (Fig. 4A) and demonstrated a significantly reduced sensitivity to PM, particularly at lower concentrations of PM (0.625 and 1.25 *μ*M) compared to the control cells (MiaPaCa-2, 5 vs. 61%, and 54 vs. 83%; Panc-1, 3 vs. 22%, and 33 vs. 70%). Similarly, to investigate the effect of a reduced expression of hTERT on the response to PM, tumor cells were transfected with hTERT siRNA for 48 h. As shown in Fig. 4B, transfection with siRNA-hTERT significantly reduced the levels of hTERT in the two cell lines (insets). A decrease in hTERT levels significantly (p<0.05) increased the sensitivity of the two cell lines to PM at concentrations that were otherwise inactive or only slightly active (MiaPaCa-2, 19.6, 23 and 72% vs. 2, 1.4 and 53%; Panc-1, 38, 42 and 52% vs. 0, 4 and 18% at 0.157, 0.312 and 0.625 *μ*M PM). Transfection with empty plasmid or non-targeting siRNA had no effect on response of cells to PM (data not shown). These data demonstrated that hTERT regulates the response to PM in pancreatic cancer cells.

### PM inhibits hTERT regulatory proteins

**Figure 5:**
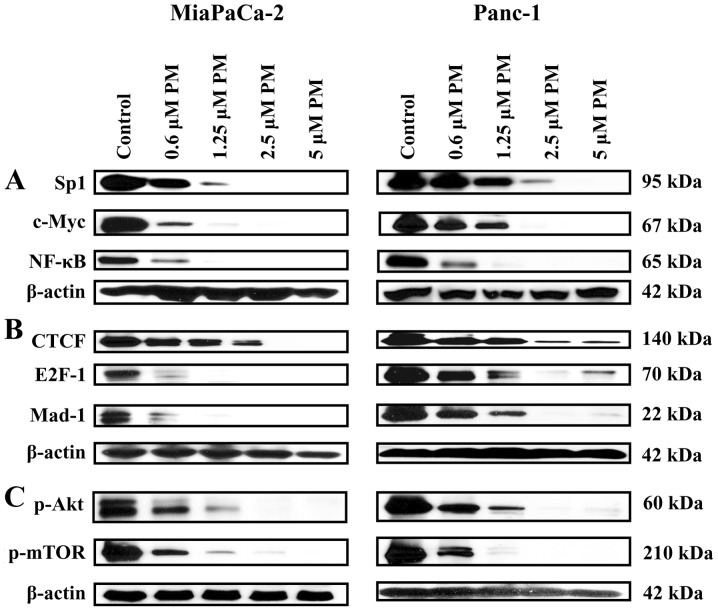
PM inhibits hTERT regulatory proteins in PDA cells. (A) MiaPaCa-2 and Panc-1 cells were treated with PM (0–5 *μ*M) for 48 h and the cell lysates were analyzed for (A) transcription factors c-Myc, Sp1 and NF-κB or (B) inhibitors of hTERT transcription (CTCF, E2F1 and Mad-1) or (C) signaling proteins p-Akt and p-mTOR by western blotting. This experiment was repeated twice. PM, pristimerin; PDA, pancreatic ductal adenocarcinoma.

Transcription factors such as c-Myc, Sp1, NF-κB and STAT-3 control the transcription of the *hTERT* gene (26,27) and post-translationally, the phosphorylation of hTERT on Ser^227^ and Ser^826^ residues by Akt is necessary for the activation of telomerase activity and the nuclear translocation of hTERT (28,29). Therefore, we assessed the effect of PM on the levels of these hTERT regulatory proteins. Treatment with PM (0.625–5 *μ*M) for 48 h partially to completely inhibited Sp1, c-Myc and NF-κB (Fig. 5A). Of note, the inhibitory factors that negatively regulate the transcription of the *hTERT* gene (CTCF, E2F1 and Mad-1) were also reduced or completely inhibited following treatment with PM (Fig. 5B). Furthermore, p-Akt and p-mTOR that post-translationally regulate hTERT were also inhibited by PM (Fig. 5C). These data demonstrated that PM downregulates hTERT expression and activity through the inhibition of transcription factors (Sp1, c-Myc and NF-κB) and signaling molecules p-Akt and p-mTOR, respectively.

## Discussion

Identification of novel agents and new targets for treating pancreatic cancer is imperative. Several studies have shown the antiproliferative and apoptosis-inducing activity of pristimerin (PM) in various types of tumor cells, including pancreatic cancer cells, through the inhibition of antiapoptotic or prosurvival pathways (9–13), providing some insights into the mode of action of PM. Telomerase, the enzyme that plays a crucial role in maintaining telomere length and chromosomal stability is activated in >90% of all human types of cancers including pancreatic cancer (22). Activated telomerase promotes tumorigenesis and tumor growth (20,21). By contrast, the inhibition of telomerase induces telomere shortening and apoptosis in cancer cells. Of all the pancreatic cancers, PDA shows the highest increase in telomerase activity (24). However, the significance of telomerase in mediating the antitumor activity of PM in PDA cells has not been investigated. Thus, the present study was undertaken to examine the role of telomerase in mediating the proapoptotic activity of PM in PDA cells. PM inhibited cell proliferation and blocked cell-cycle progression in the G1-phase. The inhibition of cell proliferation and cell cycle arrest in G1-phase by PM may have led to induction of apoptosis in the two cell lines as demonstrated by the increased binding of Annexin V and cleavage of PARP-1. These results confirm the findings of another study that also reported cell cycle arrest in G1-phase by PM in PDA cells (11).

Since cell proliferation and apoptosis are regulated by telomerase, we investigated whether inhibition of cell proliferation and induction of apoptosis correlate with the expression and telomerase activity of hTERT in tumor cells treated with PM. Specifically, analysis of hTERT mRNA by RT-PCR showed attenuation of hTERT mRNA by PM. Western blot analysis also showed a decrease in the levels of basal and phosphorylated hTERT. PM also significantly inhibited hTERT telomerase activity as measured by the TRAP assay. Thus, the inhibition of cell proliferation and induction of apoptosis by PM correlated with the inhibition of hTERT and its telomerase activity and suggested that inhibition of telomerase is part of the mechanism by which PM inhibits proliferation and induces apoptosis in PDA cells. However, further study is required to determine whether inhibition of hTERT by PM results in the shortening of telomeres and whether PM binds and degrades the RNA template of telomerase.

The present study has also demonstrated the relevance of hTERT in mediating the antitumor activity of PM in PDA cells. We found that the expression level of hTERT influenced the response of PDA cells to PM. An increase in the expression of hTERT through gene knockin rendered PDA cells more resistant to PM. On the other hand, knockdown of the expression of hTERT with siRNA hTERT increased the sensitivity of PDA cells to concentrations of PM that are otherwise inactive or only slightly active (0.156–0.625 *μ*M). These data suggested that hTERT is a potential molecular target of PM in PDA cells.

hTERT expression is driven by the binding of transcription factors c-Myc, Sp1, NF-κB and STAT-3 to their consensus sequences in hTERT promoter (26,27). Inhibition of these transcription factors by PM impacted transcription of the *hTERT* gene. We found that PM inhibited Sp1, c-Myc and NF-κB in the two PDA cell lines, suggesting that inhibition of these transcription factors is at least partially responsible for the reduced *hTERT* gene transcription and reduced hTERT protein in cells treated with PM. By contrast, various repressors of gene transcription, such as CTCF, E2F-1 and Mad-1 were also reduced or inhibited in cells treated with PM. This result suggests that inhibition of the transcription factors Sp1, c-Myc and NF-κB may be sufficient for the inhibition of hTERT expression by PM.

Post-translationally, phosphorylation of hTERT on Ser^227^ and Ser^826^ by protein kinase B/Akt is required for nuclear import and activation of its telomerase activity (28,29). The inhibition of telomerase activity in PDA cells may result from the inhibition of kinase B/Akt by PM. PM inhibited activated Akt (p-Akt). Thus, inhibition of hTERT telomerase activity may be due to the lack of phosphorylation of hTERT by Akt. hTERT is also regulated epigenetically through chromatin remodeling and DNA methylation at the hTERT promoter (30). However, whether PM impacts these epigenetic events is currently being investigated. Collectively, results of the present study identified telomerase as a potential target that may be exploited for the treatment of PDA with PM.
